# The burden of intracranial atherosclerosis on cerebral small vessel disease: A community cohort study

**DOI:** 10.1002/acn3.70005

**Published:** 2025-04-17

**Authors:** Joseph Amihere Ackah, Heng Du, Wenjie Yang, Huixing Zeng, Jason Tsz Lok Chan, Michael Lung Cheung Lo, Xiangyan Chen

**Affiliations:** ^1^ Department of Health Technology and Informatics The Hong Kong Polytechnic University Kowloon Hong Kong SAR China; ^2^ Department of Neurology Shanghai General Hospital, Shanghai Jiao Tong University School of Medicine Shanghai China; ^3^ Department of Diagnostic Radiology and Nuclear Medicine University of Maryland Baltimore Baltimore Maryland USA

## Abstract

**Objective:**

Exploring the prevalence and association between intracranial atherosclerosis (ICAS) and cerebral small vessel diseases (CSVD), this study delved beyond the current scope, utilising high‐resolution vessel wall MRI (HRVW‐MRI) to investigate how subtle changes in intracranial atherosclerotic features influence the various burdens of CSVD.

**Methods:**

Stroke‐free Chinese adult participants were recruited from our ongoing community‐based MRI cohort. HRVW‐MRI technique with a T1‐weighted 3D SPACE sequence was used to assess atherosclerotic plaque features: plaque load, degree of stenosis, remodelling index, eccentricity. A multi‐sequence MRI assessment elucidated CSVD markers, including white matter hyperintensities, lacune infarcts, microbleeds and enlarged perivascular spaces. Statistical analyses, including sensitivity and specificity tests, chi‐square, correlation and regression models were fitted to explore the association between ICAS and CSVD.

**Results:**

Of the 225 participants (mean age 64.90 ± 6.87 years) included in the study, 101 (45%) were males. Thirty‐nine participants (17.3%) presented with ICAS (8 progressive plaques and 31 were pre‐atherosclerotic). One hundred and six (47.1%) participants recorded at least one clinically significant marker of CSVD. The subtle changes (increment or decrement) in atherosclerotic features such as positive remodelling, plaque load, eccentricity, degree of stenosis and the morphology (ICAS severity) may parallelly influence the distinct markers and overall CSVD burden.

**Interpretation:**

This study demonstrates that the association between ICAS and CVSD extends beyond mere co‐existence due to shared risk factors, suggesting the presence of a dose–effect relationship between ICAS and CVSD. HRVW‐MRI could elucidate diagnostic metrics and characteristic features that reveal how ICAS impacts distinct CSVD burdens, thereby enhancing clinical decisions.

## Introduction

As the population ages and lifestyles evolve, stroke, the second global leading cause of death, has emerged as the foremost cause of mortality in China, as highlighted by the 2021 China Stroke Surveillance Report.^1^ Intracranial atherosclerosis (ICAS) is particularly responsible for nearly half (50%) of ischaemic strokes in Asian populations, a stark contrast to the 10–15% observed in Caucasians, and it further contributes to a 20% recurrence rate of stroke within a year.^2^, ^3^ Ageing can disrupt cerebral microvasculature, leading to cerebral small vessel diseases (CSVD), which may present typical neuroimaging phenotypes such as lacunar infarcts, white matter hyperintensities (WMH), cerebral atrophy, microbleeds, enlarged perivascular spaces (PVS).^4^, ^5^, ^6^ The ageing‐related pathological phenomena of ICAS and CSVD predispose the brain to various cerebrovascular events, such as strokes and other cognitive impairments.^7^, ^8^ These diseases present a significant challenge to healthcare burden.

While ICAS and CSVD are both associated with ageing and significant contributors to stroke, the precise relationship between these conditions has shown inconsistent patterns. While some studies have reported positive associations between ICAS and CSVD,^9^, ^10^ others found no significant or a mere coexistence association, suggesting that despite sharing common risk factors, atherosclerotic pathologies in the upstream cerebral large arteries may not influence the burden of CSVD.^11^, ^12^, ^13^ These varied findings have been partly attributed to the use of traditional techniques that relied solely on lumen patency and stenosis, overlooking other critical features that could elucidate the complex interplay between ICAS and CSVD.^10^, ^14^ These conventional methods often fail to provide comprehensive insights into the vessel wall pathology, plaque load, morphological and remodelling features, potentially leading to a suboptimal understanding of the underlying mechanisms of cerebrovascular diseases as well as diagnosis and treatment strategies.^3^ Fast‐forward, the application of high‐resolution vessel wall MRI (HRVW‐MRI) neuroimaging techniques has enabled the characterisation of plaque load and morphology and the identification of high‐risk features that inform prognosis and treatment strategies.^15^, ^16^ However, few studies have made use of this technique to investigate ICAS and specific phenotypes of CSVD.^10^, ^17^ Some investigations were retrospective and subjected to methodological limitations, including selection bias and suboptimal statistical power, as acknowledged by authors, whereas others did not explore in detail the quantitative metrics of arterial plaques as derived from vessel wall imaging.^9^, ^12^

In fact, for clinical application and therapeutic purposes, research must go beyond the current scope, which primarily focusses on comparing the presence of intracranial plaques with the presence or burden of small vessel disease. With advancements in high‐resolution vessel wall imaging, we can now explore with high precision how atherosclerotic features such as positive remodelling, plaque load, eccentricity, degree of stenosis and the morphology of non‐stenotic atherosclerotic plaques influence not just the overall CSVD burden but also specific subtypes and severity patterns of CSVD.^18^, ^19^ Previous investigation on the patterns of ICAS in the anterior and posterior circulation^16^ highlighted how these aforementioned plaque features could offer insights into the clinical implications of ICAS among stroke patients. It is also emphasised how some arterial segments with ICAS could undergo expansive remodelling to elude detection on the conventional time‐of‐flight magnetic resonance angiography. It is therefore clearer that these plaque features could be further explored using HRVW‐MRI to offer detailed insights into the connection between ICAS and CSVD, especially in the community‐based setting where the majority present with non‐stenotic atherosclerotic plaques.^20^

This study aimed to elucidate the relationship between ICAS and CSVD within a community‐based cohort. The study specifically explored the prevalence and association between ICAS and CSVD, delving beyond the current scope to investigate how subtle changes in intracranial atherosclerotic features, as derived from HRVW‐MRI, influence the various burdens of CSVD. A sub‐objective was to evaluate how arterial remodelling and plaque eccentricity derived from HRVW‐MRI could predict the structural features of subclinical or asymptomatic plaques. These evaluations could help understand how ICAS affects the total CSVD burden as well as the vulnerability of neuroparenchyma in developing various small vessel lesions.

## Materials and Methods

### Study design and participants

This prospective study is from an ongoing community‐based MRI project, recruiting healthy middle‐aged and older (≥45 years) Chinese adults in Hong Kong. Participants were excluded if they had a history of dementia, stroke, substance abuse, poor MRI image quality or were diagnosed with any neuropsychiatric condition using simple random sampling techniques from 2022 to 2024. The research protocol conforms to the ethical guidelines of the 1964 Declaration of Helsinki and was approved by the Ethics Committee of The Hong Kong Polytechnic University (ethics approval: HSEARS20210720002). Following a rigorous screening, 237 participants were recruited to partake in the study and underwent MRI brain scanning. Each eligible participant provided written informed consent prior to participating in the study. This study was reported according to the STROBE guidelines.^21^

### Magnetic resonance imaging acquisition

Brain imaging was conducted using a 3.0‐Tesla MRI (Siemens Medical System, Erlangen, Germany) with a 16‐channel head coil at the University Research Facility in Behavioural and Systems Neuroscience (UBSN), the Hong Kong Polytechnic University. The imaging protocol encompassed multiple sequences including a three dimensional (3D) T1‐weighted Magnetization‐Prepared Rapid Acquisition Gradient Echo (MPRAGE) sequence to assess structural changes, a 3D T2 fluid attenuated inversion recovery (FLAIR) and diffusion‐weighted imaging (DWI) sequences for detection of cerebral small vessel disease (e.g., white matter intensities and lacunes), a susceptibility‐weighted imaging (SWI) and Blood Oxygenation Level Dependent (BOLD) sequence for detection of microbleeds and a T2‐weighted Turbo Spin Echo (TSE) sequence for detection of enlarged perivascular spaces, a time‐of flight MRI (TOF‐MRI) sequence for luminal stenosis and a T1 Sampling Perfection with Application optimised Contrast using different flip angle Evolution (SPACE) sequence for plaque characteristics.

Parameters for the T1‐weighted *MPRAGE* sequence were as follows: 0.8 mm sagittal slices, flip angle = 8°, relaxation time (TR) = 2500 ms, echo time (TE) = 2.22 ms, field of view (FOV) = 256 mm × 256 mm, auto‐voxel size = 0.8 × 0.8 × 0.8 mm^3^, acceleration factor = 2, acquisition time = 6.54 minutes. T2‐weighted *TSE* sequence: 4 mm transverse slices, flip angle = 150°, TR = 6000 ms, TE = 99 ms, FOV = 220 mm × 220 mm, Auto Voxel Size = 0.3 × 0.3 × 4.0 mm^3^, acceleration factor = 2, acquisition time = 1.26 minutes. For T2‐weighted *SWI* sequence: 1.5 mm transverse slices, flip angle = 15°, TR = 27 ms, TE = 20 ms, FOV = 220 mm ×Video220 mm, Auto Voxel Size = 0.9 × 0.9 × 1.5 mm^3^, acceleration factor = 2, acquisition time = 4.54 minutes. *BOLD* sequence 1.5 mm transverse slices, flip angle = 52°, TR = 800 ms, TE = 38 ms, FOV = 208 mm × 208 mm, Auto Voxel Size = 2.0 × 2.0 × 2.0 mm^3^, acceleration factor = 8, acquisition time = 6.41 minutes. T2‐weighted *FLAIR* sequence: 0.9 mm sagittal slices, TR = 7000 ms, TE = 395 ms, FOV = 230 mm × 230 mm, Auto Voxel Size = 0.9 × 0.9 × 0.9 mm^3^, acceleration factor = 6, acquisition time = 3.46 minutes. *TOF‐MRI* sequence: 0.3 mm sagittal slices, TR = 20.3 ms, TE = 4.3 ms, FOV = 200 mm × 200 mm, Auto Voxel Size = 0.5 × 0.5 × 0.3 mm^3^, acceleration factor = 12, acquisition time = 3.51 minutes. *T1 SPACE* sequence for vessel wall imaging: 0.66 mm sagittal slices, TR = 900 ms, TE = 15 ms, FOV = 210 mm × 210 mm, Auto Voxel Size = 0.7 × 0.7 × 0.7 mm^3^, acceleration factor = 2, acquisition time = 5.56 minutes.

### Assessment of intracranial atherosclerosis

A visual assessment of brain MRI images was independently conducted using the OsiriX DICOM viewer (Pixmeo SARL, Bernex, Switzerland, version 13.0.2) in reference to established protocols from our previous investigations.^22^, ^23^, ^24^ The assessments were performed by two evaluators: a trained neuroimaging clinical investigator (J.A.A) with 6 months of experience, and a consultant neurologist (H.D.) with over 5 years of experience in vessel wall MRI assessments. Both evaluators were blinded to the clinical data to ensure unbiased analysis. Any discrepancies in the results were judicated by a senior neurologist (X. C), who was blinded to the initial findings. Intracranial atherosclerotic plaque was detected as an eccentric thickening in the vessel‐wall, with or without luminal stenosis, as detected using HRVW‐MRI with an isotropic 3D T1‐weighted SPACE sequence and 3D TOF‐MRI sequence. Plaques were assessed in the major intracranial arteries, including the intracranial portions of the bilateral internal carotid arteries, middle cerebral arteries, anterior cerebral arteries, posterior cerebral arteries, basilar arteries and the intracranial segments of the bilateral vertebral arteries. For each plaque, 3D HRVW‐MRI images were reconstructed perpendicular to the vessel axis in 0.6 mm thick. The cross‐sectional area with the most substantial plaque was identified as the lesion site for measurement. Proximal to the lesion site, a normal thin‐walled segment with no plaque noted in the cross section was designated as the reference site as adopted in previous study.^18^ The nearest plaque‐free cross section distal to the plaque lesion was regarded as the alternative reference site in cases where there was no proximal plaque‐free segment. Based on histopathology‐validated imaging features,^16^, ^25^, ^26^ the severity of identified plaques was classified as either pre‐atherosclerotic plaque or progressive plaque. Pre‐atherosclerotic plaques were identified by their smaller size, higher lipid content and minimal contrast enhancement, appearing as high signal intensity on T1‐weighted images with relatively smooth thickened walls. Progressive plaques were characterised by larger, irregular structures with necrotic cores, calcifications, significant contrast enhancement and intraplaque haemorrhage, showing heterogeneous signal intensity on T1‐ and T2‐weighted images, along with marked vessel wall thickening and luminal narrowing.

### Quantitative assessment on HRVW‐MRI

**Figure 1:**
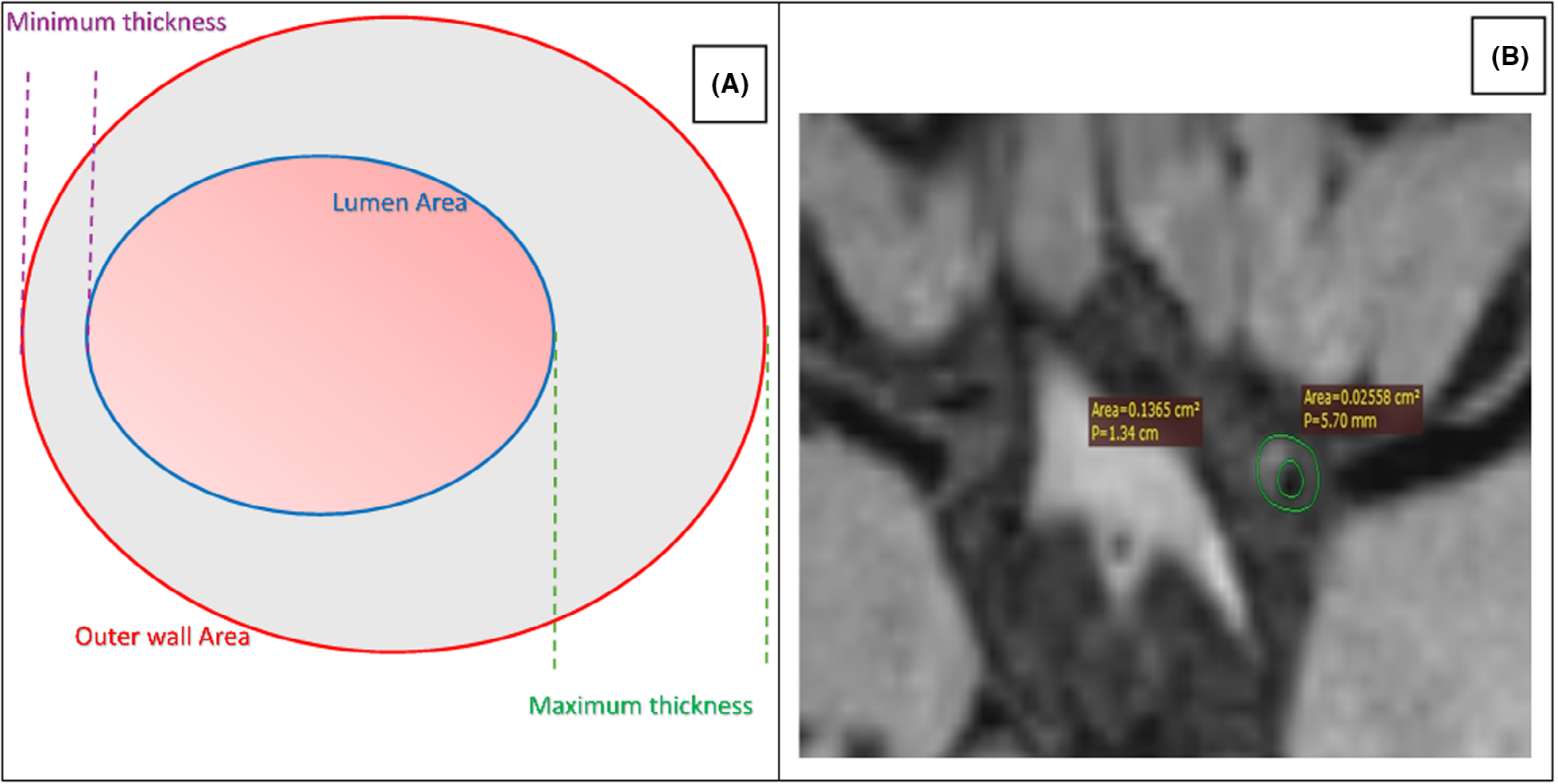
(A) Schematic representation of the key components involved in the measurement and quantification of plaque features. (B) Representative image of a plaque detected and quantified using High‐Resolution Vessel Wall MRI (HRVW‐MRI).

Subtle vessel wall atherosclerotic changes visualised on HRVW‐MRI were evaluated and quantified. Quantitative assessments of ICAS (shown in Fig. 1) involved measuring the outer wall area (OWA) and lumen area (LA) of both the lesion and reference sites.^16^ Plaque area was calculated as OWA_lesion_ minus LA_lesion_. Plaque load or plaque burden was calculated by (plaque area/OWA_lesion_) × 100%. The degree of stenosis at the lesion site was calculated as (1‐LA_lesion_/LA_reference_) × 100%. The remodelling index (RI) was determined as dividing the outer wall area (OWA_lesion_/OWA_reference_). Remodelling patterns were classified as follows: positive remodelling for RI >1.05, intermediate or no remodelling for 0.95 ≤ RI ≤ 1.05, and negative remodelling for RI <0.95.^18^ The maximum wall thickness (WT_max_) and minimum wall thickness (WT_min_) were measured, and the eccentricity index was calculated using the formula (WT_max_ – WT_min_)/WT_max_. Plaques were identified as having either eccentric or concentric wall thickening if the eccentricity index was ≥0.5 or <0.5, respectively.^19^

### Assessment of cerebral small vessel disease

**Figure 2:**
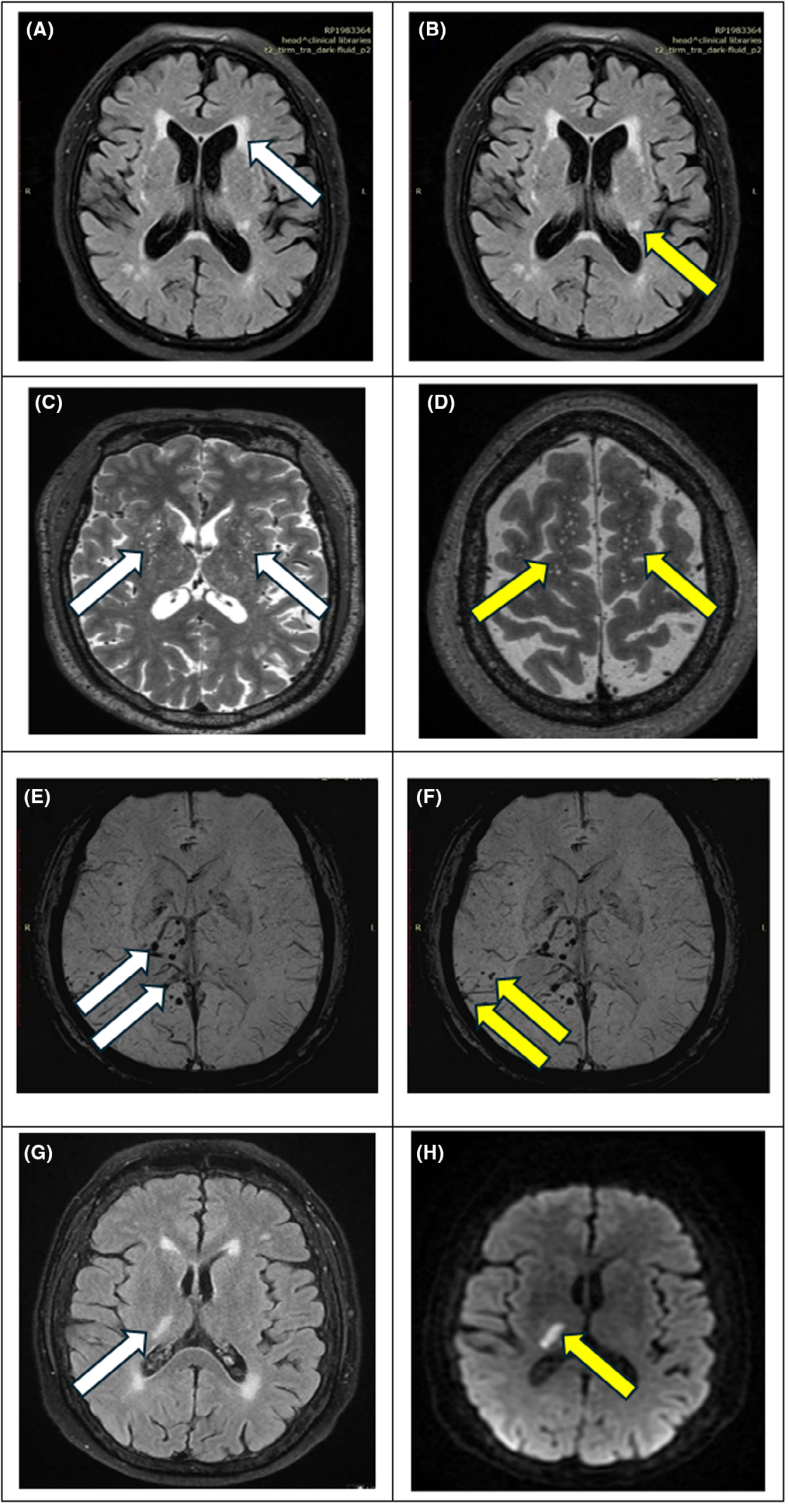
(A) Axial brain MRI on FLAIR sequence showing periventricular white matter hyperintensities (white arrow). (B) Axial brain MRI on FLAIR sequence showing deep subcortical white matter hyperintensities (yellow arrow). (C) Axial brain MRI T2‐weighted sequence showing perivascular spaces in the basal ganglia (white arrows). (D) Axial brain MRI T2‐weighted sequence showing perivascular spaces in the centrum semiovale (yellow arrows). (E) Axial brain MRI susceptibility‐weighted imaging sequence showing deep cerebral microbleeds (white arrows). (F) Axial brain MRI susceptibility‐weighted imaging sequence showing lobar cerebral microbleeds (yellow arrows). (G) Axial brain MRI FLAIR sequence demonstrating an acute infarct in the posterior limb of the right internal capsule (white arrow). (H) Axial brain MRI diffusion‐weighted imaging sequence demonstrating an acute infarct in the posterior limb of the right internal capsule (yellow arrow).

The OsiriX DICOM viewer (Pixmeo SARL, Bernex, Switzerland, version 13.0.2) was used to perform the neuroimaging visual assessment of the phenotypes or biomarkers of CSVD (Fig. 2) by two raters (J.A.A. and H.Z.), who were blinded to clinical data. CSVD assessments were performed in reference to standardised criteria set in the STRIVE‐1^6^ and the recently advanced version STRIVE‐2.^27^ WMH were identified as lesions that appear brighter in the white matter on T2‐weighted FLAIR images. A previously validated 8‐rating scale was adopted to assess the combined and exclusive periventricular and deep WMH.^28^, ^29^ Four categories for WMH burden based on the severity scores were established as follows: absent (grade 0), mild (grade 1–2), moderate (grade 3–5), and severe (grade 6–8). Lacunes were identified as fluid‐filled cavities, resembling cerebrospinal fluid on all sequences, with diameters ranging from 3 to 15 mm and having a round or oval shape. Cerebral microbleeds (CMBs), including those in lobar and deep regions, were defined as 2 to 10 mm round or oval hypointense lesions on T2 susceptibility‐weighted images. Perivascular spaces (PVS) were characterised as small punctate (<3 mm) or linear hyperintensities on T2 images, with those in the basal ganglia and centrum semiovale. The lacunes, CMBs and ePVS were based on count of lesions identified. The total CSVD score or burden, as designed by Wardlaw's group, ranged from 0 to 4, with one point assigned for the presence of lacunes, a moderate‐to‐severe WMH burden, the presence of CMBs and moderate‐to‐severe basal ganglia PVS (*n* > 10).

### Clinical covariates

A trained research assistant with a background in neurology collected the demographic and clinical data. This process involved not only gathering self‐reported information from community‐based participants but also directly measuring certain clinical parameters. These measurements included vital signs, such as heart rate and blood pressure, in addition other relevant health metrics, ensuring a comprehensive and accurate data set. Variables recorded include age, gender, blood pressure, history of hypertension, diabetes, smoking, drinking and hyperlipidemia. Hypertension was identified as a previous diagnosis of hypertension or a recording of systolic blood pressure (SBP) ≥ 140 mmHg and/or diastolic blood pressure (DBP) ≥90 mmHg.^30^ Diabetes mellitus was characterised by a prior diagnosis or a fasting glucose level of 7.0 mmol/L or higher, and/or a random blood glucose level of 11.1 mmol/L or higher.^31^ Body mass index (BMI) was measured based on the weight and height of participants.^32^ The classifications of smoking and drinking were based on participants' self‐report and were both defined as continuous or cumulative smoking and consumption of alcohol for more than 6 months in a lifetime, respectively, as adopted by Zheng and colleagues.^22^ Data protection protocols and confidentiality were strictly adhered to.

### Statistical analysis

The SPSS 29.0 software package (SPSS, Inc.) was used for all statistical analysis. Continuous variables are presented as either the mean with standard deviation (SD) or the median with interquartile range, while categorical variables are shown as percentages and frequencies. To compare continuous variables, a Mann–Whitney *U* test and independent t‐test were used. Categorical variables were compared using the chi‐squared test. Pearson's linear correlation analysis systematically elucidated the relationships between the four quantitative metrics of ICAS—plaque load, degree of stenosis, remodelling index and eccentricity—and various imaging phenotypes of CSVD. These phenotypes included the lacune infarcts, WMH in both periventricular and deep regions, deep and lobar CMBs, enlarged PVS in the basal ganglia and centrum semiovale, and total CSVD burden.

Pierson's chi‐squared analysis was performed to explore the association between the severity of ICAS and the different burdens of CSVD imaging phenotypes. In a further analysis, the relationships between CSVD burden and intracranial atherosclerotic plaque features (Remodelling index, plaque burden, degree of stenosis and eccentricity) were examined using Pierson's chi‐squared correlation analysis. The area under the curve values as well as sensitivity and specificity values for plaque features in indicating the plaque burden and severity were also determined.

Multivariate ordinal regression analysis was carried out to evaluate the independent associations between intracranial arterial plaque load as well as severity of ICAS and the various burdens of CSVD including the overall CSVD burden (as defined by Wardlaw).^6^ Two models were used for adjustments: Model 1 adjusted for age; Model 2 adjusted for age, gender and vascular risk factors (hypertension, diabetes mellitus, hyperlipidaemia, smoking, drinking and BMI) as well as other cerebral small vessel disease markers.

Statistical significance was considered at a *p*‐value of less than 0.05.

## Results

### Baseline characteristics of participants

Following the MRI brain imaging, 225 individuals from our community‐based MRI cohort were considered eligible for this study after excluding 12 out of the 337 participants due to the following factors: 7 had poor MRI image resolution, and 5 had incomplete MRI scans. Consequently, a complete data set of 225 participants (mean age 64.90 ± 6.87 years), including 101 males (45%), was analysed. Among the 225 participants, 68 (30.2%) have hypertension, 85 (37.8%) have hyperlipidaemia, 23 (10.2%) have diabetes, 12 (5.3%) are active smokers and 21 (9.3%) consume alcohol. None of the participants had experienced a stroke or had any known heart conditions.

### The prevalence of ICAS and its morphological and plaque features

Thirty‐nine participants (17.3%) presented with ICAS and were relatively older than those without ICAS. Other clinical baseline parameters were comparable between the two groups, as shown in Table 1. Of the 39 participants with ICAS, only eight showed features of progressive plaques, with the highest degree of stenosis and plaque load reaching 66.56% and 89.6%, respectively, while the remaining 31 were pre‐atherosclerotic. Among this relatively healthy stroke‐free cohort, ICAS was predominantly observed in the posterior circulation, with plaque counts distributed as follows: 16 in the right VA, 8 in the left VA, 5 in the BA, and 1 in the left PA. In the anterior circulation, plaques were recorded as follows: 3 in the left ACA, 2 in the right MCA, 2 in the right ICA, 1 in the left ICA, and 1 in the left MCA.

**Table 1:** Baseline characteristics of included participants.

Parameter	Total participants (*n* = 225)	Participants with ICAS (*n* = 39)	Participants without ICAS (*n* = 186)	*p* value
Age (mean ± SD, years)	64.90 ± 6.87	67.26 ± 6.03	64.4 ± 6.9	0.018^*^
Systolic BP (mean ± SD)	129.88 ± 16.91	133.64 ± 16.00	129.10 ± 17.03	0.127
Diastolic BP (mean ± SD)	79.31 ± 10.25	79.49 ± 8.03	79.27 ± 10.68	0.904
Heart Pulse rate (mean ± SD)	73.05 ± 11.22	75.56 ± 12.50	72.52 ± 10.89	0.124
BMI (mean ± SD)	23.68 ± 3.19	23.71 ± 2.93	23.67 ± 3.25	0.947
MAP (mean ± SD)	96.17 ± 11.50	97.54 ± 9.15	95.88 ± 11.93	0.413
Gender [Male, *n* (%)]	101 (45.0)	17 (43.6)	84 (45.2)	0.858
Hypertension, [Yes, *n* (%)]	68 (30.2)	12 (30.8)	56 (30.1)	0.935
Hyperlipidemia, [Yes, *n* (%)]	85 (37.8)	19 (48.7)	66 (35.5)	0.122
Diabetes, [Yes, *n* (%)]	23 (10.2)	5 (12.8)	18 (10.6)	0.556
Smoking, [Yes, *n* (%)]	12 (5.3)	3 (7.7)	9 (4.8)	0.471
Drinking, [Yes, *n* (%)]	21 (9.3)	1 (2.6)	20 (10.8)	0.110
Cerebral small vessel disease				
WMH [Yes, *n* (%)]	180 (80)	39 (100)	141 (75.8)	<0.001^*^
pWMH [Yes, *n* (%)]	129 (57.3)	33 (84.6)	96 (51.6)	<0.001^*^
dWMH [Yes, *n* (%)]	177 (78.7)	37 (94.9)	140 (75.3)	<0.001^*^
Lacune infarct [Yes, *n* (%)]	29 (12.9)	11 (28.2)	18 (9.7)	0.002^*^
ePVS [Yes, *n* (%)]	164 (72.9)	39 (100)	125 (67.2)	<0.001^*^
Basal ganglia (BG)	35 (15.6)	8 (20.5)	27 (3.8)	<0.001^*^
Centrum semiovale (CSO)	73 (32.4)	2 (5.2)	71 (38.2)	<0.001^*^
Combined BG + CSO	117 (52)	29 (74.4)	88 (47.3)	<0.001^*^
Microbleeds [Yes, *n* (%)]	33 (14.7)	9 (23.1)	24 (12.9)	0.102
Lobar CMB's [Yes, *n* (%)]	18 (8)	7 (17.9)	11 (5.9)	0.012
Deep CMB's [Yes, *n* (%)]	11 (4.8)	2 (5.1)	9 (4.8)	0.939
Total CSVD burden				
0	119 (52.9)	7 (17.9)	112 (60.2)	
1	62 (27.6)	11 (28.2)	51 (27.4)	
2	28 (12.4)	13 (33.4)	15 (8.1)	<0.001^*^
3	12 (5.3)	7 (17.9)	5 (2.7)	
4	4 (1.8)	1 (2.6)	3 (1.6)	

BP, blood pressure; ICAS, intracranial atherosclerosis; SD, standard deviation.

* *p* < 0.05.

In the assessment of the remodelling index among the 39 participants with ICAS, 17 arterial segments exhibited positive remodelling, with the majority noted in the posterior circulation. Negative remodelling was observed in 14 segments, while eight segments demonstrated intermediate or no remodelling. Regarding eccentricity, 35 plaques (89.7%) were classified as eccentric, while four plaques (10.3%) were concentric.

As shown in Table 2, both positive remodelling and eccentric plaques are highly sensitive indicators of plaque load, degree of stenosis, plaque area, lumen area, and the overall severity of ICAS. These indicators demonstrated excellent area under the curve (AUC) values, along with high sensitivity and specificity associated with the parameters assessed.

**Table 2:** Sensitivity and specificity analysis (receiver operator characteristics curve).

Test result variable(s)	Area	Std. error	Asymptotic Sig.	Asymptotic 95% confidence interval		Cut of point	Sensitivity (%)	Specificity (%)
				Lower bound	Upper bound			
Positive remodelling								
Lumen area	0.955	0.014	0.000	0.928	0.982	0.016	88.2	91.0
Severity of ICAS	0.955	0.014	0.000	0.929	0.982	n/a	100	89.4
Plaque load%	0.952	0.014	0.000	0.923	0.980	68.72	88.2	91.0
Plaque area	0.963	0.012	0.000	0.939	0.987	0.086	94.1	91.8
CSVD burden	0.781	0.057	0.000	0.670	0.892	n/a	88.0	56.2
Eccentricity								
Lumen area	0.990	0.006	0.000	0.978	1.002	0.016	88.7	98.4
Severity of ICAS	0.992	0.005	0.000	0.982	1.001	n/a	100	98.0
Plaque load%	0.996	0.003	0.000	0.991	1.002	68.72	94.3	99.0
Plaque area	0.992	0.006	0.000	0.981	1.003	0.086	85.7	98.4
CSVD burden	0.783	0.045	0.000	0.694	0.872	n/a	83.0%	60.0%

### The severity of ICAS and the different burdens of CSVD markers

Out of the 225 participants, enlarged PVS, lacune infarcts, WMH, and cerebral microbleeds were detected in 164 (72.9%), 29 (12.9%), 180 (80%), and 33 (14.7%) participants, respectively. Participants with ICAS have a significantly higher prevalence of WMH, lacune infarcts, enlarged PVS, and cerebral microbleeds when compared with those without ICAS (Table 1). According to the Wardlaw score, 106 (47.1%) participants presented with clinically significant scores or moderate to severe burdens of CSVD. The distribution of participants based on the Wardlaw CSVD score was as follows: 62 individuals (27.6%) scored 1, 28 individuals (12.4%) scored 2, 12 individuals (5.3%) scored 3 and 4 individuals (1.8%) scored 4.

Pearson's chi‐squared analysis in Table 3 revealed significant correlations between the severity of ICAS—characterised by pre‐atherosclerotic or progressive plaque features—and the various burdens of CSVD markers such as WMH (*χ* ^2^ = 34.13, *df* = 6, *p* < 0.001), ePVS (*χ* ^2^ = 42.55, *df* = 6, *p* < 0.001), lacune infarcts (*χ* ^2^ = 11.73, *df* = 4, *p* = 0.019) and total CSVD burden (*χ* ^2^ = 50.75, *df* = 8, *p* < 0.001). However, there were no significant correlations observed between the severity of ICAS and the burden of cerebral microbleeds (*χ* ^2^ = 3.23, *df* = 4, p = 0.520).

**Table 3:** Chi‐square correlations between the severity of ICAS and the burdens of CSVD.

	Participants with different severity of ICAS			*χ* ^2^ (correlation)	*df*	*p*‐value
	No ICAS (*n* = 186)	Pre‐ICAS (*n* = 31)	Prog‐ICAS (*n* = 8)			
WMH burden, *n* (%)						
Absent	45 (24.20)	0 (0.00)	0 (0.00)			
Mild	125 (67.20)	20 (64.52)	8 (100.00)	34.132	6	<0.001^*^
Moderate	16 (8.60)	9 (29.03)	0 (0.00)			
Severe	0 (0.00)	2 (6.45)	0 (0.00)			
Combined EPVS burden, *n* (%)						
0	61 (32.80)	0 (0.00)	0 (0.00)			
≤10	71 (38.17)	6 (19.35)	2 (25.00)	42.547	6	<0.001^*^
11–20	40 (21.50)	15 (48.39)	5 (62.50)			
≥21	14 (7.53)	10 (32.26)	1 (12.50)			
CMBs burden, *n* (%)						
0	162 (87.10)	24 (77.42)	6 (75.00)			
1	16 (8.60)	5 (16.13)	1 (12.50)	3.232	4	0.520
≥2	8 (4.30)	2 (6.45)	1 (12.50)			
Lacune burden, *n* (%)						
0	168 (90.32)	23 (74.19)	5 (62.50)			
1	16 (8.60)	7 (22.58)	3 (37.50)	11.727	4	0.019^*^
≥2	2 (1.08)	1 (3.23)	0 (0.00)			
Total CSVD burden, *n* (%)						
0	112 (60.22)	6 (19.35)	1 (12.50)			
1	51 (27.42)	8 (25.81)	3 (37.50)	50.751	8	<0.001^*^
2	15 (8.06)	9 (29.03)	4 (50.00)			
3	5 (2.69)	7 (22.58)	0 (0.00)			
4	3 (1.61)	1 (3.23)	0 (0.00)			

CMBs, cerebral microbleeds; EPVS, enlarged perivascular space; WMH, white matter hyperintensities.

* *p* < 0.05. Total CSVD scores 0‐4 indicate the level of severity burden in increasing order.

### Associations between ICAS plaque features and various CSVD markers

Pearson's chi‐squared correlation analysis for categorical variables showed that the presence of ICAS is significantly associated with the presence of WMH (*χ* ^2^ = 11.79, *df* = 1, *p* < 0.001), lacunae infarcts (*χ* ^2^ = 9.86, *df* = 1, *p* = 0.002), ePVS (*χ* ^2^ = 17.55, *df* = 1, *p* < 0.001), and total CSVD burden (*χ* ^2^ = 41.68, *df* = 4, *p* < 0.001), but showed no significant association with the presence of cerebral microbleeds (*χ* ^2^ = 2.67, *df* = 1, *p* = 0.102). Pearson's linear correlation analysis (Table 4) revealed that plaque features, including plaque load, degree of stenosis, remodelling index and eccentricity, showed statistically significant positive correlations with the presence of periventricular WMH, deep subcortical WMH, and the severity of WMH, the presence and total count of lacune infarcts, the presence and total count of ePVS in addition to the presence of ePVS in both the basal ganglia and centrum semiovale, as well as the total burden of CSVD. Notably, the presence of CMBs showed a significant association exclusively with the remodelling index. Lobar CMBs were significantly associated with key quantitative metrics of ICAS, including plaque load, remodelling index, and eccentricity, except for the degree of stenosis. In contrast, deep CMBs exhibited no significant associations with any metrics of ICAS.

**Table 4:** Correlations between ICAS plaque features and CSVD imaging phenotypes.

CSVD	Plaque load^1^	Degree of stenosis^2^	Remodelling index^3^	Eccentricity^4^	*p* value^1^	*p* value^2^	*p* value^3^	*p* value^4^
WMH severity [*r* (95% CI)]	0.318 (0.195, 0.431)	0.233 (0.106, 0.535)	0.312 (0.189, 0.426)	0.308 (0.185, 0.422)	<0.001	<0.001	<0.001	<0.001
pWMH [*r* (95% CI)]	0.265 (0.139, 0.382)	0.209 (0.080, 0.330)	0.247 (0.120, 0.366)	0.269 (0.144, 0.387)	<0.001	0.002	<0.001	<0.001
dWMH [*r* (95% CI)]	0.176 (0.047, 0.300)	0.148 (0.017, 0.273)	0.179 (0.049, 0.302)	0.166 (0.036, 0.291)	0.008	0.027	0.007	0.012
Lacune infarct [*r* (95% CI)]	0.216 (0.088, 0.338)	0.182 (0.053, 0.306)	0.238 (0.111, 0.358)	0.208 (0.079, 0.330)	<0.001	0.006	<0.001	0.002
Lacune count [*r* (95% CI)]	0.208 (0.079, 0.330)	0.140 (0.009, 0.266)	0.245 (0.118, 0.364)	0.197 (0.068, 0.320)	0.002	0.036	<0.001	0.003
ePVS [*r* (95% CI)]	0.274 (0.148, 0.390)	0.227 (0.099, 0.347)	0.267 (0.141, 0.385)	0.272 (0.146, 0.389)	<0.001	<0.001	<0.001	<0.001
ePVS count [*r* (95% CI)]	0.415 (0.301, 0.518)	0.315 (0.192, 0.428)	0.421 (0.307, 0.523)	0.403 (0.287, 0.507)	<0.001	<0.001	<0.001	<0.001
BG + CSO ePVS [*r* (95% CI)]	0.258 (0.132, 0.376)	0.224 (0.096, 0.345)	0.250 (0.123, 0.368)	0.262 (0.136, 0.379)	<0.001	<0.001	<0.001	<0.001
Microbleeds [*r* (95% CI)]	0.118 (−0.013, 0.245)	−0.005 (−0.135, 0.126)	0.149 (0.018, 0.274)	0.105 (−0.027, 0.232)	0.077	0.943	0.026	0.118
CMB's count [*r* (95% CI)]	0.053 (−0.079, 0.182)	−0.029 (−0.159, 0.102)	0.077 (−0.054, 0.206)	0.038 (−0.093, 0.168)	0.431	0.662	0.249	0.572
Lobar CMB's [*r* (95% CI)]	0.179 (0.050, 0.303)	0.057 (−0.075, 0.186)	0.205 (0.076, 0.327)	0.162 (0.032, 0.287)	0.007	0.398	0.002	0.015
Deep CMB's [*r* (95% CI)]	0.005 (−0.126, 0.136)	−0.048 (−0.178, 0.083)	0.022 (−0.109, 0.152)	0.004 (−0.127, 0.135)	0.940	0.469	0.745	0.951
Total CSVD burden	0.388 (0.271, 0.493)	0.285 (0.160, 0.401)	0.400 (0.284, 0.504)	0.373 (0.255, 0.480)	<0.001	<0.001	<0.001	<0.001

### Multivariate ordinal regression analysis between severity of ICAS, plaque load, and CSVD burdens

Table 5 shows that the severity of ICAS is an independent predictor of WHM burden [OR = 2.826, 95% CI (1.586–5.038), *p* < 0.001], lacune burden [OR = 2.573, 95% CI (1.374–4.816), *p* = 0.003], ePVS burden [OR = 3.983, 95% CI (2.340–6.780), *p* < 0.001], as well as total CSVD burden [OR = 3.714, 95% CI (2.221–6.209), *p* < 0.001].

**Table 5:** Multivariate Ordinal regression on the correlations between the plaque burden and severity of ICAS and the imaging features of CSVD.

	ICAS‐involved arteries					
	None		Plaque load		Severity of ICAS	
	*p* value			*p* value		*p* value
WMH burden (absent, mild, moderate, severe)						
Crude OR (95% CI)	1.000		1.023 (1.013–1.033)	<0.001*	2.826 (1.586–5.038)	<0.001*
Model‐1 OR (95% CI)	1.000		1.022 (1.011–1.032))	<0.001*	2.75 (1.490–5.078)	0.001*
Model‐2 OR (95% CI)	1.000		1.020 (1.008–1.032)	<0.001*	2.447 (1.194–5.013)	0.015*
EPVS burden (0, ≤10, 11–20, ≥21)						
Crude OR (95% CI)	1.000		1.025 (1.017–1.035)	<0.001*	3.983 (2.340–6.780)	<0.001*
Model‐1 OR (95% CI)	1.000		1.025 (1.016–1.035)	<0.001*	3.641 (1.906–6.621)	<0.001*
Model‐2 OR (95% CI)	1.000		1.020 (1.010–1.029)	<0.001*	3.111 (1.765–5.479)	<0.001*
Lacune burden (0, 1, >2)						
Crude OR (95% CI)	1.000		1.017 (1.006–1.028)	0.002*	2.573 (1.374–4.816)	0.003*
Model‐1 OR (95% CI)	1.000		1.015 (1.004–1.026)	0.007*	2.452 (1.297–4.641)	0.006*
Model‐2 OR (95% CI)	1.000		1.012 (1.000–1.024)	0.050*	2.171 (1.070–4.402)	0.032*
Total CSVD burden (0, 1, 2, 3, 4)						
Crude OR (95% CI)	1.000		1.025 (1.017–1.034)	<0.001*	3.714 (2.221–6.209)	<0.001*
Model‐1 OR (95% CI)	1.000		1.023 (1.015–1.032)	<0.001*	3.508 (2.090–5.894)	<0.001*
Model‐2 OR (95% CI)	1.000		1.025 (1.016–1.034)	<0.001*	2.545 (1.415–4.577)	0.002*

Model‐1 adjusted for age. Model‐2 adjusted for age, gender, and vascular risk factors (hypertension, diabetes mellitus, hyperlipidaemia, smoking, drinking, and BMI as well as CSVD phenotypes).

CI, confidence interval; EPVS, enlarged perivascular space; OR, odds ratio; WMH, white matter hyperintensities.

* *p* < 0.05.

Additionally, plaque load is also an independent predictor of WHM burden [OR = 1.023, 95% CI (1.013–1.033), *p* < 0.001], lacune burden [OR = 1.017, 95% CI (1.006–1.028), *p* = 0.002], ePVS burden [OR = 1.025, 95% CI (1.017–1.035), *p* < 0.001], as well as total CSVD burden [OR = 1.025, 95% CI (1.017–1.034), *p* < 0.001].

After adjusting for age in *Model 1* and further adjusting for age, gender, vascular risk factors, as well as other cerebral small vessel disease markers in *Model 2*, both the severity of ICAS and plaque load remained statistically significant predictors of CSVD burdens.

### Inter and intra‐rater reliability of ICAS and CSVD assessments

The inter‐rater reliabilities of the burden of ICAS and each CSVD phenotype were evaluated separately. The weighted kappa of ICAS burden was 0.918 [95% CI (0.761–1.075), *p* < 0.001]; the weighted kappa for WMH burden was 0.881 [95% CI (0.735–1.027), *p* < 0.001]; the weighted kappa for ePVS burden was 0.928 [95% CI (0.828–1.029), *p* < 0.001]; the weighted kappa for lacune burden was 0.940 [95% CI (0.833–1.048), *p* < 0.001]; the weighted kappa for cerebral microbleeds was 0.947 [95% CI (0.849–1.045), *p* < 0.001]. Each rater attained an excellent intra‐rater reliability based on the intra‐class reliability coefficients (>9.0 each).

## Discussion

This study reveals that among relatively healthy, stroke‐free individuals in a community‐based setting, the prevalence of ICAS is 17.3%, with the majority exhibiting non‐stenotic pre‐atherosclerotic plaques. The findings reveal that 47.1% of individuals in the community‐based cohort exhibit at least one marker indicative of a clinically significant or moderate‐to‐severe burden of CSVD. The presence of ICAS is shown to be associated with various markers of cerebral small vessel diseases characterised by the presence and burden of WMH, including periventricular WMH and deep subcortical WMH, the presence and burden of lacune infarcts, the presence and burden of ePVS, including both the basal ganglia and centrum semiovale ePVS. Intracranial atherosclerotic plaque load or burden is shown to serve as an all‐encompassing semiquantitative gauge of total brain atherosclerosis.^33^ This study agrees that plaque load and its association with CSVD could accurately reflect the health of the brain's primary arteries, even the non‐stenotic ones.

Previous studies have questioned the direct association between ICAS and CSVD, suggesting that these cerebral pathologies may merely co‐exist due to shared risk factors such as hypertension or diabetes.^11^, ^12^, ^13^ In a meta‐analysis, Zhang and colleagues reported non‐significant association between ICAS and WHM, the commonest marker of CSVD.^13^ However, recent studies challenge this perspective by revealing robust positive associations that indicate a more intricate relationship beyond simple co‐occurrence.^9^, ^10^, ^14^, ^34^ Noted among these prior studies are some methodological challenges and research gaps that have been addressed in this current study. While some studies included patients with embolic strokes of undetermined source with limited generalisabilty, others lacked the statistical power necessary for robust parameter comparisons. Previous HRVW‐MRI studies have suggested that a higher burden of ICAS across multiple vascular beds is associated with an increased burden of CVSD.^9^, ^10^ However, some of these studies reported potential selection bias due to retrospective design and did not explore in detail the association of other quantitative metrics of arterial plaques as derived from vessel wall imaging. In other studies, the evaluation of ICAS using CT scans provided insights into the volume and severity of intracranial arterial calcification, while some estimated the percentage of stenosis via MRA to inform therapy and management. However, atherosclerotic surrogates derived from CT and MRA capture only a portion of intracranial atherosclerotic plaque, potentially leading to inaccurate prevalence scores due to overestimation or underestimation, particularly in the context of arterial remodelling.^14^, ^34^, ^35^

For instance, in this current study, approximately 45% of the atherosclerotic arterial segments demonstrated positive remodelling, a phenomenon frequently observed in the posterior cerebral circulation.^16^ It is shown that positive remodelling, which can elude the identification of ICAS when using traditional techniques such as TOF‐MRA, is associated with higher plaque load, degree of stenosis, plaque area, lumen area, and the overall increase in the severity of ICAS. Again, although plaques are widely known to present as eccentric lesions in vessel assessment, the eccentricities of plaques observed in this study revealed that ICAS could present as concentric plaques on HRVW‐MRI. Until now, this observation had only been previously reported in stroke patients by our research team in previously published histology‐validated MRI assessment of atherosclerotic plaques in postmortem brain specimens.^36^ Evaluating these plaque features necessitates the use of HRVW‐MRI that offers a more comprehensive vessel‐wall analysis that extends beyond the capabilities of TOF‐MRA and CT angiography. Using HRVW‐MRI, the current prospective study critically incorporated important atherosclerotic features, such as plaque load, eccentricity, degree of stenosis, remodelling index and severity based on plaque morphology, to provide a more accurate and comprehensive reflection of the complex interplay between ICAS and CVSD. The findings from the multivariate ordinal regression analysis provide compelling evidence that subtle features of intracranial atherosclerosis such as the severity of ICAS and plaque load are significant independent predictors of various CSVD burdens. The persistence of these associations after adjusting for multiple confounding factors indicates that the observed relationships are robust and not merely attributable to shared risk factors. This strengthens the argument for a direct link between ICAS and CSVD.

It is crucial to understand that the variability in findings concerning the relationship between ICAS and various CSVD imaging phenotypes might be due to the diffuse nature of CSVD, and certain imaging phenotypes may stem from indirect vascular pathological alterations.^37^ In a previous study, we reported a strong link between medial calcifications in intracranial arteries and WMH,^35^ suggesting a shared underlying mechanism between ICAS and CSVD. This current study distinguished WMH into periventricular and deep subcortical WMH and further showed that intracranial atherosclerosis is associated with both phenotypes of WMH. Although the underlying mechanism is not fully understood, previous studies have linked the development of WMH to hypoperfusion cascades of atherosclerosis‐induced ischaemia.^38^ Based on the findings from the current study, one can posit that atherosclerotic vascular changes may also be associated with glymphatic impairment, evident on MRI as enlarged perivascular spaces in both centrum semiovale and basal ganglia, as well as lacunar infarcts, both of which are phenotypes of CSVD.^10^, ^39^ While deep CMBs are typically linked to hypertension‐induced arterial stiffness, lobar CMBs are often associated with cerebral amyloid angiopathy, which involves amyloid deposition compromising vessel integrity.^40^, ^41^, ^42^ Therefore, the correlation between the remodelling index and the presence of lobar CMBs, suggest that ICAS‐induced remodelling may predominantly facilitate amyloid deposition, as compared to hypertension‐induced stiffness in deep cerebral regions. Although previous studies^18^, ^41^, ^43^ suggest that arterial wall stiffness and remodelling are key mechanisms in the development of microbleeds and CSVD, this finding warrants further investigation.

The real‐world applicability and implementation of HRVW‐MRI requires careful consideration of several factors. Although with the availability of an MRI with a high‐field strength MRI (preferably ≥3 T) and the applicable sequences (T1‐weighted 3D SPACE), HRVW‐MRI could be implemented,^44^ only a few specialised centres are performing this technique.^45^ Based on the findings from a recent study on the implementation of vessel wall imaging in an academic medical facility utilising the ‘reach, effectiveness, adoption, implementation, maintenance (RE‐AIM) framework’,^45^ workflow challenges involved setting up examination protocols and the duration of scans. Additionally, interpreting HRVW‐MRI images requires specialised expertise in vessel wall neuroradiology, utilising widely used neuroimaging software like RadiAnt and OsiriX DICOM viewers. Despite the challenges, the potential benefits of incorporating HRVW‐MRI into clinical practice are substantial.^46^, ^47^ By providing detailed insights into vessel wall pathology, HRVW‐MRI can enhance precision diagnosis and inform primary prevention strategies, allowing for earlier intervention, targeted lifestyle modifications and personalised management plans.^48^, ^49^ It is therefore important for clinicians to adopt the HRVW‐MRI as a routine diagnostic and screening tool for the assessment of ICAS.

This study has some limitations, including its cross‐sectional design, which prevents the determination of the directional relationship between ICAS and CSVD. While this approach does not track the progression of ICAS and CSVD severity over time, it provides a valuable snapshot that enhances our understanding of their current burden and interplay. Future longitudinal studies could build on these findings to further elucidate the temporal dynamics between these conditions. The study population was restricted to Chinese adults, which may limit the generalisability of its findings. However, unlike previous studies that focused solely on older adults with mean ages over 80 years, this study included both middle‐aged and elderly adults. Consequently, the results may provide a more accurate reflection of the ageing population, encompassing a broader age range. The study is also free from selection bias given its prospective robust recruitment strategies of random selection of Chinese participants from different communities in Hong Kong. The validity of the current findings is supported by the community‐based design and the systematic approach for assessing the cSVD biomarkers, in addition to the use of validated semiquantitative methods to evaluate ICAS across different intracranial vascular beds using internationally recognised methods. These factors collectively represent a significant strength of our study.

In conclusion, this study demonstrates that the association between ICAS and CVSD extends beyond mere co‐existence due to shared risk factors, suggesting the presence of a dose‐effect relationship between ICAS and CVSD. HRVW‐MRI has the potential to elucidate diagnostic metrics and characteristic that reveal how ICAS affects distinct CSVD burdens, thereby enhancing clinical decision‐making.

## Conflict of Interest

The authors declare that they have no conflict of interest.

## Author Contribution

For the assessment of brain MRI images, J.A.A., a neuroimaging PhD candidate, received 6 months of specialised training in brain and vessel wall MRI image assessment from W.Y. and X.C., who are experienced neurologists and researchers with decades of experience. H.Z., A newly qualified neurologist, received similar training prior to the image assessment. Additionally, H.D., a consultant neurologist, and W. Y., a clinical neurologist with neuroradiology expertise, bring over 5 years of research experience in brain image assessment to the study. The training was based on established protocols as detailed in our previous studies.^16^, ^23^, ^24^, ^48^ The training included both theoretical and practical components, focusing on high‐resolution vessel wall imaging techniques and the interpretation of imaging results. Conceptualization and design of research (J.A.A., H.D and X.C); Data curation (J.A.A. and J.T.L.C.); Formal analysis (J.A.A. and X.C.); Funding acquisition (X.C); Investigation (J.A.A., H.D., X.C., W.Y., Y.C., H.Z., J.T.L.C., and M.L.C.L); Methodology (J.A.A, H.D., X.C., H.D., Y.C., H.Z., J.T.L.C., and M.L.C.L); Project administration (J.A.A, X.C., H.Z., J.T.L.C., and M.L.C.L); Resources (X.C., W.Y., Y.C., J.T.L.C., and M.L.C.L); Software (J.A.A and X.C.); Supervision (X.C.); Validation (X.C., J.A.A., H.D., and W.Y.); Roles/Writing – original draft (J.A.A); and Writing – review & editing (J.A.A., X.C., W.Y., and H.Z.).

## Data Availability

The data sets generated and/or analysed during the current study are available from the corresponding author (X.C) on reasonable request.
